# Psychological Impact and Quality of Life in Adults With Tinnitus: A Cross-Sectional Study

**DOI:** 10.7759/cureus.51976

**Published:** 2024-01-09

**Authors:** Abdullah Musleh, Amal Kaaled H Alharthy, Manar Yahya M Alzahrani, Seba Ahmed Bin Maadhah, Ibrahim Ali Al Zehefa, Raghad Yahya AlQahtani, Ibtihal Sultan M Alshehri, Faisal Bayram A Alqahtani, Khalid Ali M Asiri, Abdullah Ali Almushari

**Affiliations:** 1 Otolaryngology - Head and Neck Surgery, King Khalid University Hospital, Khamis Mushait, SAU; 2 College of Medicine, Ibn Sina National College for Medical Studies, Jeddah, SAU; 3 College of Medicine, King Khalid University, Abha, SAU; 4 College of Medicine, Batterjee Medical College, Jeddah, SAU

**Keywords:** mental problems, anxiety, depression, quality of life, tinnitus

## Abstract

Background: Tinnitus may directly or indirectly influence professional, personal, and leisure activities and disrupt family and social relationships in severe cases. This study aimed to explore the impact of tinnitus on the quality of life (QoL) and psychological well-being among Saudi Arabian adults.

Methods: This study used a cross-sectional design to collect data from adults experiencing tinnitus. Data collection took place between September 5, 2023 and October 7, 2023 at King Khalid University, Abha, Saudi Arabia. The study involved adults aged 18 to 65 with tinnitus. Participants were recruited from audiology clinics. A multi-stage stratified random sampling method was used to recruit the study participants. The severity of tinnitus handicap was assessed using the 25-item Tinnitus Handicap Inventory (THI) questionnaire. THI consists of 25 items divided into functional, emotional, and catastrophic subscales. The total score ranges from 0 to 100, with higher scores indicating greater tinnitus-related handicap. THI scores are categorized into 0-16, 18-36, 38-56, 58-76, and 78-100.

Results: A total of 163 participants were included in this study. The largest age group category was 18-29 years, comprising 57.05% (n = 93) of the sample. The study included a slightly higher percentage of female participants (65.03%, n = 106). More than three-fifths (61.96% ) of the participants were from the Western Region (n = 101), 91.40% (n = 149) were Saudi nationals, 55.21% (n = 90) were single, 67.5% (n = 110) had university education, 46.0% (n = 75) had income less than 500 SAR, 44.78% (n =73) was employed, and 74.23% (n = 121) did not smoke. Tinnitus hurts emotions and QoL. The largest proportion of the studied patients with tinnitus faced catastrophic handicaps (24.5%, n = 40), and nearly equal proportions experienced mild and moderate handicaps (23.3%, n = 38 and 23.9%, n =39, respectively). Severe handicap was reported in 15.3% (n = 25), while a slight effect was reported in (12.9%, n = 21). Tinnitus affected their hearing (36.81%, n = 60); interfered with daily life (33.74%, n = 55), social activities (30.06%, n = 49), social relationships (29.45%, n = 48), and concentration (37.42%, n = 61); caused fatigue (38.04%, n = 62) and sleep disturbances (36.81%, n = 60); and prevented them from enjoying life (25.77%, n = 42). Tinnitus caused anger (47.85%, n = 78), confusion (42.94%, n = 70), anxiety (43.56%, n = 71), feeling unsafe (33.74%, n = 55), desperation (36.81%, n = 60), frustration (30.06%, n = 49), being upset (38.04%, n = 62), experiencing depression (30.67%), and challenges in coping with stress (31.29%). There was a significant association between the THI score and region of residence (p = 0.02), income (p = 0.041), occupation (p = 0.013), and smoking (p = 0.014).

Conclusions: Our research underscored the profound impact of tinnitus on the QoL among adults in Saudi Arabia. A significant portion of the studied patients faced catastrophic handicaps, emphasizing the severity of the condition. These findings underscore the multifaceted and far-reaching consequences of tinnitus, highlighting the need for comprehensive support and management strategies tailored to the unique sociodemographic factors influencing individuals' experiences.

## Introduction

Despite significant advances in the field of medicine, tinnitus continues to pose a perplexing challenge to clinicians, both from the scientific and clinical perspectives. This audiological condition is far from being a rare occurrence in routine clinical practice. Tinnitus, often characterized by the perception of sound within the ears or head, is often described as a ringing, buzzing, or whistling sound [1].

Episodes of chronic tinnitus can be intermittent or constant, lasting three months or more and at least five minutes each time. Subjective tinnitus, on the other hand, is more common and is caused by an aberrant brain activity unrelated to outside noises [2]. Presbycusis and noise-induced hearing loss are two prevalent forms of hearing loss that are frequently associated to tinnitus. Tinnitus can either occur in isolation or as a part of a symptom complex related to specific otological diseases, such as ototoxicity, Meniere's disease, noise-induced sensorineural hearing loss, and presbycusis [3]. Numerous research evaluated how tinnitus affected patients' lives and overall quality of life (QoL). Tinnitus can interfere with social and familial bonds; affect one's career, personal, and recreational pursuits; and, in extreme circumstances, even result in suicidal thoughts [4]. While the association between tinnitus and impaired QoL is recognized, insufficient evidence remains to pinpoint the specific characteristics contributing to this association. These include factors related to the tinnitus complaint and the outcomes of audiological evaluations, such as the type and extent of hearing loss [5].

The prevalence of tinnitus is Saudi Arabia is high. In a study conducted at King Khalid University in Abha, Saudi Arabia, focusing on investigating the prevalence and determinants of tinnitus among health science students. A total of 400 students aged 18-30 participated in the study. Noteworthy findings include 51.8% of students experiencing bilateral tinnitus, with 44.7% describing buzzing sounds and 21.1% indicating hissing sounds [4]. In another study involving 400 students aged 18-30, 28.5% reported positive findings related to tinnitus. Notably, 51.8% of students experienced bilateral tinnitus, with 44.7% reporting buzzing sounds, 21.1% indicating hissing, and 10.5% perceiving pulsating sounds. The prevailing characteristics of the reported tinnitus included moderate loudness and intermittent occurrences among the majority of students [6].

The term "quality of life" (QoL) refers to "an individual's perception of their position in life" [7]. This subjective assessment takes place "in the context of the culture and value systems in which they live, as well as in relation to their goals, expectations, standards, and concerns" [8]. It is important to highlight that the QoL of people living in the region has undergone a significant impact in recent times, coinciding with the global coronavirus disease (COVID-19) pandemic. Both the general population and healthcare workers have experienced disruptions, with consequences extending to health and well-being [9-11]. Tinnitus has a detrimental effect on the QoL [12,13]. We hypothesized that tinnitus significantly and adversely affects the psychological well-being and overall QoL among adults in Saudi Arabia. We anticipate that individuals with tinnitus in Saudi Arabia may experience lower QoL scores. Despite the widespread global occurrence of tinnitus, there is a noticeable lack of research addressing its psychological implications and specific impact on the QoL among adults in Saudi Arabia. Recognizing the importance of unique cultural and societal factors in Saudi Arabia is crucial for a comprehensive understanding of how tinnitus impacts individuals in this region. Consequently, this study aims to address this research gap by exploring the QoL implications of tinnitus among adults in Saudi Arabia.

## Materials and methods

Study design

This study employed a cross-sectional design to collect data from a sample of adults experiencing tinnitus. The data collection took place between September 5, 2023 and October 7, 2023 at at King Khalid University, Abha, Saudi Arabia.

Study participants

The study included adults aged 18 to 65 experiencing tinnitus, and the participants were recruited from audiology clinics. To ensure a representative sample size, a multi-stage stratified random sampling method was applied. Geographical locations (urban and rural) served as the basis for the stratification of audiology clinics. Within each stratum, one health facility was randomly selected. The participants were then chosen from each healthcare facility using a simple random sampling technique. A list of participants was compiled, and the selection process was carried out using a random sequence generator.

Sample size calculation

The G*power software (Franz Faul, Universität Kiel, Germany) was utilized to determine that a minimum sample size of 134 is required, taking into account the following assumptions: a power of 95%, an alpha error of 5%, an effect size of 0.3, and mean Tinnitus Handicap Inventory (THI) score of 36.6 ± 19.7 [13]. The specified size of the mean THI score is 0.3. In this case, an effect size of 0.3 suggests a moderate effect. They voluntarily participated in the study without any incentives. The participants received comprehensive information about the study's objectives, procedures, potential risks, benefits, and their right to withdraw at any time.

Data collection

Data were gathered through the use of validated self-report questionnaire. The questionnaire gathers sociodemographic information on the participants, including age groups ranging from 18 to 65 years, gender (male or female), region of residence (Eastern, Middle, Northern, Southern, or Western Region of Saudi Arabia), nationality (Saudi or non-Saudi), marital status (divorced, married, or single), education level (ranging from preparatory to postgraduate), monthly income (categorized into income ranges below 5000 SAR, between 5000 and 10,000 SAR, and above 15,000 SAR), occupation (working, not working, retired, or student), and smoking habits (yes or no). The severity of tinnitus handicap was assessed using the validated Arabic version of the THI questionnaire [14]. This questionnaire, which has been widely adopted, includes 25 items divided into functional (11 items), emotional (nine items), and catastrophic (five items) subscales. Each item provides three response options (0 = none, 2 = sometimes, 4 = always), and the total THI score is obtained by summing all the responses. The possible total score for the THI ranges from 0 to 100, with higher scores indicating a greater level of handicap resulting from tinnitus. THI scores fall into specific categories: 0-16 signifies "no or slight handicap," 18-36 corresponds to "mild" handicap, 38-56 represents "moderate" handicap, 58-76 indicates "severe" handicap, and a score falling in the range of 78-100 is classified as "catastrophic handicap" [15-17].

Data confidentiality and analysis

Access to the data was restricted to authorized researchers only. Participation in the study was entirely voluntary, and the participants could opt to decline or withdraw from the study at any time without facing any adverse consequences. Stringent security measures were implemented to safeguard the acquired data. Only the principal investigator had access to the data, and appropriate data protection protocols were followed to preserve participants' information. We used IBM SPSS Statistics for Windows, version 26 (released 2019; IBM Corp., Armonk, New York, United States) to analyze data. Descriptive statistics were utilized to summarize the demographic characteristics of the sample. Number and percentages were used to describe categorical variables. A chi-square test was used to test the association between categorical variables; in the case of violation of its assumption, Fisher's exact and Montecarlo tests were used instead.

Ethical considerations

Informed consent forms were provided, and the participants were asked to sign them before participating in the study. All collected data were kept strictly confidential. Personal identifying information of participants was coded and stored separately from the research data to ensure anonymity. The research proposal was approved by the King Khalid University Research Ethics Committee (IRB number ECM#2023-3004).

## Results

The participants were relatively evenly distributed across various age groups. The largest age group was 18-29 years, comprising 57.10% (n = 93) of the sample. The distribution gradually decreased with increasing age, with the smallest group being individuals aged 60-65 years (4.90%, n = 8). The study included a slightly higher percentage of female participants (65.03%, n = 106) compared to male participants (34.96%, n = 57). Most participants were from the Western Region (61.69%, n = 101), while the Northern Region had the fewest participants (3.68%, n = 6). The vast majority of participants were Saudi nationals (91.40%, n =149), with a smaller percentage being non-Saudi (8.59%, n = 14). The sample comprised individuals with diverse marital statuses. More than half of the participants were single (55.21%, n = 90), followed by married individuals (36.20%, n = 59) and divorced individuals (8.59%, n = 14). A significant proportion of the participants had a university education (67.50%, n = 110), while a smaller percentage had secondary education (20.86%, n =34). Primary education represented only 0.61% of the participants (n = 1). The remaining education levels have a relatively low representation in the sample. The income distribution varies, with the highest percentage falling in the "less than 500 SAR" category (46.01%, n = n = 75). The "between 5,000 and 15,000 SAR" and "more than 15,000 SAR" categories had 36.81% (n = 60) and 17.20% (n = 28) of participants, respectively. Approximately two-fifths of the participants were employed (44.79%, n = 73), followed by students (35.89%, n = 58). A smaller percentage were retired (7.98%, n = 13), and some were not currently working (11.66%, n = 19). About three-fourths of the participants did not smoke (74.23%, n = 121), while 25.77% (n = 42) were smokers (Table 1).

**Table 1:** Demographic characteristics of the study participants

Studied variables (n = 163)		Frequency	Percent (%)
Age in years	18-29	93	57.06
	30-39	30	18.40
	40-49	20	12.27
	50-59	12	7.36
	60-65	8	4.91
Gender	Male	57	34.97
	Female	106	65.03
Region	Eastern Region	13	7.98
	Middle Region	12	7.36
	Northern Region	6	3.68
	Southern Region	31	19.02
	Western Region	101	61.96
Nationality	Non-Saudi	14	8.59
	Saudi	149	91.41
Marital status	Divorced	14	8.59
	Married	59	36.20
	Single	90	55.21
Education	Post-graduate	16	9.82
	Preparatory	2	1.23
	Primary	1	0.61
	Secondary	34	20.86
	University	110	67.48
Income	Less than 500 SAR	75	46.01
	Between 5,000 and 15,000 SAR	60	36.81
	More than 15,000 SAR	28	17.18
Occupation	Working	73	44.79
	Don’t work	19	11.66
	Retired	13	7.98
	Student	58	35.58
Smoking	No	121	74.23
	Yes	42	25.77

**Figure 1:**
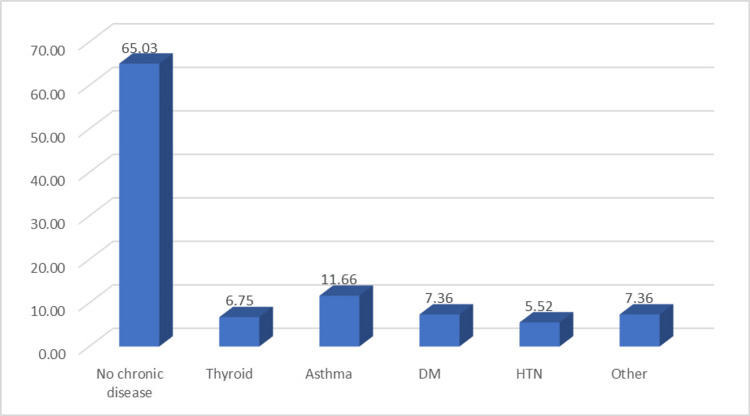
Distribution of chronic diseases among the study participants

Figure 1 illustrates the distribution of chronic diseases among the study participants, shedding light on the prevalence of various health conditions within the population having tinnitus. The majority of participants (65.03%, n =106) reported having no chronic diseases. Thyroid conditions were observed in 6.75% (n = 11) of the participants, while asthma was noted in 11.66% (n = 19) of the cases; diabetes mellitus (DM) was reported by 7.36% (n = 12) of the participants. Hypertension (HTN) was identified in 5.52% (n = 9) of the cases. An additional 7.36% (n = 12) of participants reported other chronic diseases.

**Figure 2:**
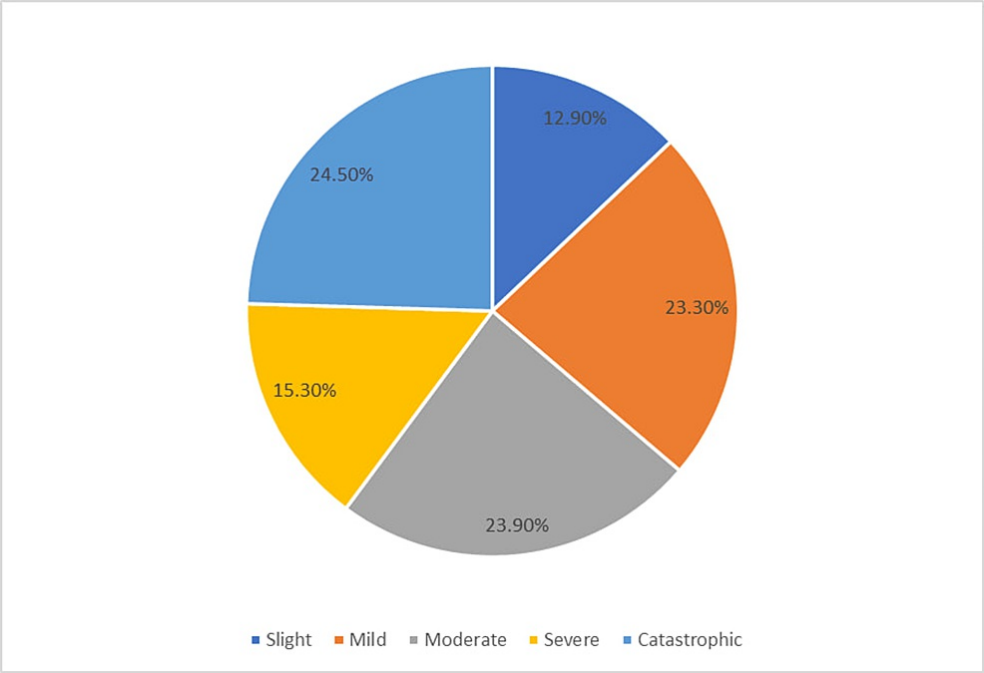
Distribution of the studied population based on their quality of life

Figure 2 shows that the largest proportion of the studied patients with tinnitus faced catastrophic handicap (24.5%, n =40) and nearly equal proportions experienced mild and moderate handicap (23.3%, n = 38 and 23.9%, n =39, respectively). Severe handicap was reported in 15.3% (n = 25), while a slight effect was reported in 12.9% (n = 21) (Figure 2).

**Figure 3:**
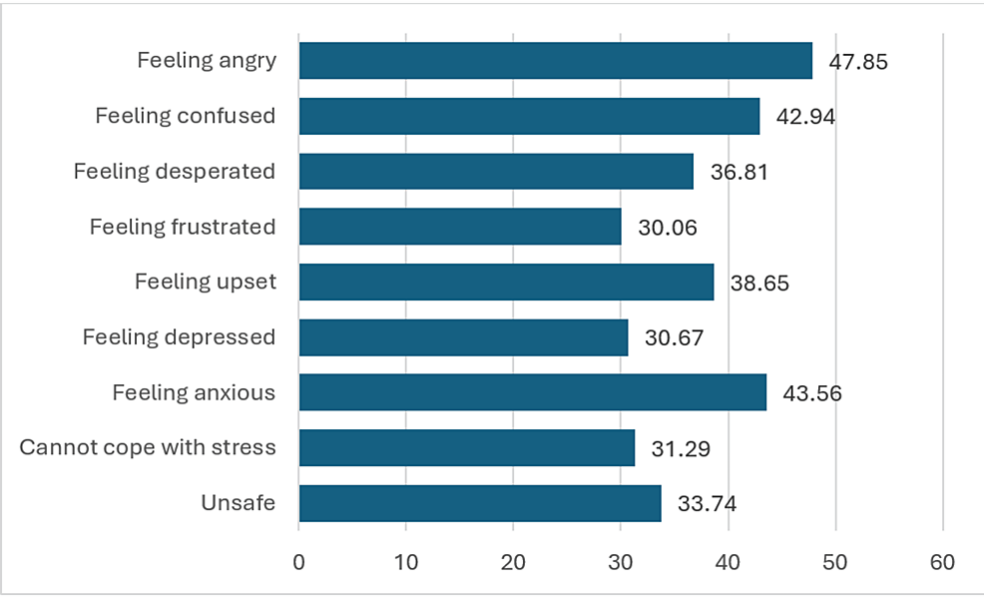
Effect of tinnitus on the participants' feelings

Tinnitus has a notable impact on various emotional and psychological aspects of individuals' well-being. The participants reported elevated levels of anger (47.85%, n = 78), confusion (42.94%, n = 70), anxiety (43.56%, n = 71), and feeling unsafe (33.74%, n = 55). In addition, a significant portion expressed feelings of desperation (36.81%, n = 60), frustration (30.06%, n = 49), being upset (38.65%, n = 63), and experiencing depression (30.67%, n = 50). Notably, a substantial proportion reported challenges in coping with stress (31.29%, n = 51) (Figure 3).

**Figure 4:**
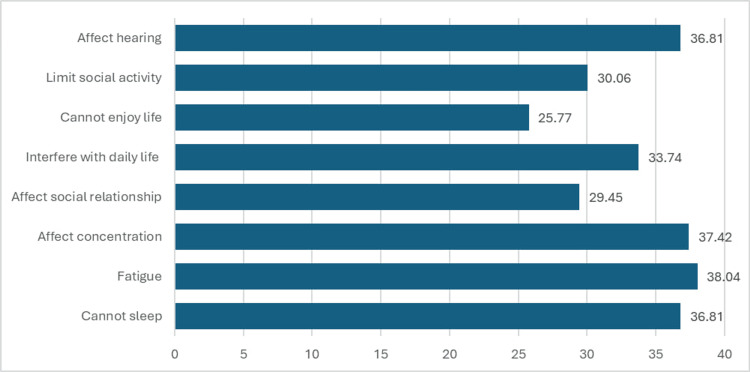
Effect of tinnitus on the quality of life

A substantial proportion reported that tinnitus affects their hearing (36.81%) and interferes with daily life (33.74%). Many individuals also noted that tinnitus has an impact on their social activities (30.06%), social relationships (29.45%), and concentration (37.42%). Furthermore, a notable percentage of participants reported experiencing fatigue (38.04%) and sleep disturbances (36.81%). In addition, a significant portion expressed that tinnitus prevents them from enjoying life (25.77%) (Figure 4).

Table 2 describes the association between different studied sociodemographic factors and tinnitus handicap inventory. There was a significant association between the region of residence (p = 0.02), income (p = 0.041), occupation (p = 0.013), and smoking (0.014).

**Table 2:** Factors associated with tinnitus handicap inventory MCP: Monte Carlo p test

Variables		Slight		Mild		Moderate		Severe		Catastrophic	
		n	%	n	%	n	%	n	%	n	%
Age	18-29	13	65.0	25	65.8	23	59.0	12	48.0	19	47.5
	30-39	2	10.0	5	13.2	7	17.9	6	24.0	10	25.0
	40-49	2	10.0	4	10.5	6	15.4	2	8.0	6	15.0
	50-59	0	0.0	2	5.3	3	7.7	5	20.0	2	5.0
	60-65	3	15.0	2	5.3	0	0.0	0	0.0	3	7.5^ MCP^
P value		0.187									
Region	Eastern Region	3	15.0	6	15.8	1	2.6	1	4.0	1	2.5
	Middle Region	4	20.0	3	7.9	1	2.6	2	8.0	2	5.0
	Northern Region	1	5.0	2	5.3	0	0.0	0	0.0	3	7.5
	Southern Region	4	20.0	9	23.7	10	25.6	6	24.0	2	5.0
	Western Region	8	40.0	18	47.4	27	69.2	16	64.0	32	80.0
P value		0.020^ MCP^									
Gender	Male	6	30.0	10	26.3	16	41.0	6	24.0	18	45.0
	Female	14	70.0	28	73.7	23	59.0	19	76.0	22	55.0
P value		0.265									
Nationality	Non-Saudi	2	10.0	5	13.2	4	10.3	1	4.0	2	5.0
	Saudi	18	90.0	33	86.8	35	89.7	24	96.0	38	95.0
P value		0.651^ MCP^									
Marital status	Divorced	2	10.0	4	10.5	3	7.7	1	4.0	4	10.0
	Married	4	20.0	11	28.9	13	33.3	13	52.0	18	45.0
	Single	14	70.0	23	60.5	23	59.0	11	44.0	18	45.0
P value		0.405^ MCP^									
Education	Post graduate	0	0.0	3	7.9	4	10.3	2	8.3	7	17.5
	Preparatory	1	4.8	0	0.0	0	0.0	1	4.2	0	0
	Primary	1	4.8	0	0.0	0	0.0	0	0.0	0	0
	Secondary	7	33.3	12	31.6	5	12.8	3	12.5	7	17.5
	University	11	52.4	23	60.5	30	76.9	19	79.2	26	65
P value		0.050^ MCP^									
Monthly income in thousand	Less than 5,000 SAR	13	65.0	23	60.5	18	24	9	36.0	12	30.0
	Between 5 and 15 SAR	6	30.0	10	26.3	16	26.7	13	52.0	15	37.5
	More than 15,000 SAR	1	5.0	5	13.2	5	18.5	3	12.0	13	32.5
P value		0.041									
Occupation	Clerk	6	30.0	11	28.9	18	46.2	12	48.0	24	60.0
	Don’t work	2	10.0	5	13.2	5	12.8	5	20.0	2	5.0
	Retired	2	10.0	0	0.0	4	10.3	2	8.0	5	12.50
	Student	10	50.0	22	57.9	12	30.8	5	20.0	9	22.50
	Teacher	0	0.0	0	0.0	0	0.0	1	4.0	0	0.0
P value		0.013^MCP^									
Smoking	No	16	80.0	35	92.1	25	64.1	15	60.00	29	72.5
	Yes	4	20.0	3	7.9	14	35.9	10	40.00	11	27.5
P value		0.014									

## Discussion

Patients experiencing tinnitus can exhibit diverse levels of annoyance, impacting their QoL. It is crucial to distinguish between the intensity of the tinnitus signal and the severity of the symptom, including the distress it causes in patients' lives. These two factors play distinct roles in understanding and addressing the challenges associated with tinnitus [13]. In the specific context of our research endeavor, our primary objective was to conduct a thorough and comprehensive assessment of the QoL experienced by individuals grappling with the challenges of tinnitus. To accomplish this multifaceted evaluation, we strategically employed the THI, a widely recognized and meticulously designed tool. The purpose of incorporating the THI was to gain a nuanced understanding of how tinnitus influences and affects various facets of individuals' overall well-being and daily life experiences.

The main study findings

There was a spectrum of tinnitus severity within the study group, with a significant proportion dealing with either severe or moderate levels of handicap. Furthermore, the study discovered significant correlations between sociodemographic variables, such as residence region, income, occupation, and smoking habits with severity scores on the THI. The findings highlight the complex nature of tinnitus, emphasizing the critical need for a multifaceted approach to addressing its multifaceted effects on individuals' overall well-being.

The study disclosed that 24.5% of the participants encountered catastrophic THI, significantly impacting sleep and daily activities. In addition, 15.3% experienced severe THI, while 39.9% reported moderate levels, 23.3% mild, and 12.9% sluggish THI. The audible ringing, seldom masked, substantially affected daily routines. These findings underscore the imperative for effective tinnitus management strategies to address the diverse severity levels and associated disruptions experienced by individuals. Lower catastrophic THI score was reported by Ukaegbe et al. [13]. They found that the THI grades among patients with tinnitus was as follows: slight (32.3%), mild (19.1%), moderate (20.6%), severe (13.2%), and catastrophic (14.7%).

Associated factors with the THI score

We did not find significant association between age and THI score. Similarly, Meric et al. [18] found no association between age and tinnitus-related effect on the QoL. On the other hand, Hiller and Goebel [19] reported increased annoyance in older patients, contrasting our results. This difference may be due to the lack of equal presentation of each age group in the mentioned studies.

Research on the impact of gender on the prevalence of tinnitus has yielded conflicting results. Some studies indicate a slightly higher prevalence in females [20], while others suggest a higher prevalence in males [21], although statistical significance is often lacking. In our study, where women were in the majority, participant sex did not show an association with the THI score. This aligns with the broader spectrum of studies that have failed to establish a significant correlation between gender and tinnitus severity. The complexity of tinnitus, influenced by various factors, underscores the need for continued exploration to better understand its multifaceted nature.

Of note, a large sector of the participants reported that tinnitus affects their hearing. On the other hand, Savastano [22] used the same evaluation tool for the THI that that more severe hearing loss died not show a association with the severity of tinnitus. This reliance on subjective data may contribute to the observed contradiction between our findings and those of Savastano, highlighting the potential influence of subjective perceptions in understanding the relationship between hearing loss and tinnitus severity.

Effect of tinnitus on the QoL

It is well established that tinnitus can negatively affect the QoL [23,24], especially in the physical and psychological domains. The study reveals that tinnitus significantly impacts individuals' lives, affecting their hearing, daily activities, social relationships, concentration, fatigue, sleep disturbances, and enjoyment of life [23]. Hoof et al. [4] examined the association between tinnitus-specific and health-related quality of life (HRQoL) questionnaires. Eighty-five patients with tinnitus completed five questionnaires: Tinnitus Sample Case History Questionnaire (TSCHQ), Tinnitus Functional Index (TFI), THI, short version of World Health Organization Quality of Life (WHOQOL-BREF), and the eight-item Short-Form (SF-8). Findings showed a negative and strong correlation between tinnitus questionnaires and the SF-8, with over half of the variability in the SF-8 scores explained by the TFI and THI. A strong negative regression was also found between the WHOQOL-BREF and the THI and TFI. This indicates a negative association between the QoL and tinnitus. These findings emphasize the broad-ranging impact of tinnitus on both the physical and social aspects of individuals' lives, underlining the need for comprehensive support and management strategies to address these challenges.

Effect of tinnitus on mental health

The majority of existing evidence on the relationship between tinnitus and mental health stems from relatively small-scale clinical studies. These studies consistently reveal a significant association between tinnitus and psychiatric disorders, with reported rates ranging from 45% to 78% of tinnitus patients meeting the criteria for a lifetime psychiatric diagnosis [25-27]. Among tinnitus patients, a range of clinical studies has reported prevalence rates of diagnosed current depressive disorders, falling within the range of 33-74% [25,26,28-30], and the prevalence of anxiety disorders ranging from 29-49% [29,30]. Our findings indicate that tinnitus has a significant impact on emotional and psychological well-being, resulting in heightened levels of anger, confusion, anxiety, and feelings of unsafeness, desperation, frustration, upsetness, depression, and difficulties in coping with stress. Similarly, the Gutenberg Health Study investigated the correlation between tinnitus and depression, anxiety, or somatization in a large population-based cohort. Out of 8,539 participants, 28.0% had tinnitus, and depression/anxiety/somatic symptom disorders were significantly higher among those with tinnitus compared to those without [31]. However, it is noteworthy that certain studies have not revealed a particularly elevated occurrence of psychiatric symptoms within their tinnitus patient samples [32]. These findings underscore the profound psychological effects of tinnitus, indicating that it can lead to a range of negative emotions and mental states, including anger, anxiety, and feelings of unsafety. The study highlights the importance of addressing the emotional well-being of individuals with tinnitus and developing strategies to mitigate these emotional consequences.

Implication of this research

This research holds significant implications for both clinical practice and public health initiatives in the realm of tinnitus. By uncovering the varied severity levels of tinnitus and identifying influential sociodemographic factors, such as region of residence, income, occupation, and smoking, the findings provide clinicians with valuable insights to tailor personalized treatment plans. Moreover, the study underscores the importance of a comprehensive approach, emphasizing the need for interventions that go beyond clinical measures and consider the broader context of an individual's life. The use of the THI as an assessment tool suggests its relevance in routine clinical evaluations, offering a practical avenue to measure the impact of tinnitus on patients' overall QoL. Furthermore, these insights contribute to raising public awareness about the multifaceted nature of tinnitus, reducing associated stigmas, and encouraging timely intervention for improved patient outcomes.

Strengths and limitations

This study significantly advances our understanding of the QoL implications of tinnitus among adults in Saudi Arabia, addressing a notable gap in research. The adoption of a diverse sample through a multi-stage stratified random sampling method enhances the study's generalizability. The utilization of a validated questionnaire for assessing tinnitus handicap ensures standardized and reliable results. However, the study's cross-sectional design limits the ability to make causal inferences. In addition, potential selection bias may arise from recruiting participants in audiology clinics, and reliance on self-reported data, particularly regarding hearing impairment, poses a limitation. The lack of in-depth explorations of regional variations further acknowledges certain constraints in the study.

## Conclusions

Our research underscored the profound impact of tinnitus on the QoL among adults in Saudi Arabia. A significant portion of the studied patients faced catastrophic handicap, emphasizing the severity of the condition. Emotional and psychological aspects were significantly affected, with participants reporting heightened levels of anger, confusion, anxiety, and feelings of being unsafe. Desperation, frustration, upset feelings, and depression were also prevalent among respondents, indicating the far-reaching consequences of tinnitus.

The adverse effects of tinnitus, as illustrated, demonstrate the emotional toll it takes on individuals. Many participants reported challenges in coping with stress, emphasizing the broader impact on mental well-being. The multifaceted nature of tinnitus's influence on various aspects of daily life is highlighted. A substantial percentage noted adverse effects on hearing, daily activities, social engagement, relationships, concentration, fatigue, sleep patterns, and the ability to enjoy life. These findings underscore the need for comprehensive support and management strategies for individuals grappling with tinnitus. In addition, significant associations between certain sociodemographic factors and tinnitus handicap inventory were identified. The region of residence, income, occupation, and smoking demonstrated statistically significant correlations. These insights provide a nuanced understanding of how sociodemographic factors may contribute to the varying experiences of individuals with tinnitus.
